# Is Gene Transcription Involved in Seed Dry After-Ripening?

**DOI:** 10.1371/journal.pone.0086442

**Published:** 2014-01-20

**Authors:** Patrice Meimoun, Ernest Mordret, Nicolas B. Langlade, Sandrine Balzergue, Sandrine Arribat, Christophe Bailly, Hayat El-Maarouf-Bouteau

**Affiliations:** 1 UMR 7622, UPMC Univ. Paris 06, CNRS, Bat C 2 ème étage, 4, place Jussieu, 75005 Paris, France; 2 Laboratoire Interactions Plantes-Microorganismes, INRA UMR 441, CNRS, UMR 2594, BP 52627, Chemin de Borde Rouge Auzeville, 31326 Castanet Tolosan, France; 3 Unité de Recherche en Génomique Végétale (URGV), UMR INRA 1165 - CNRS 8114 - UEVE, F-91057 Evry Cedex, France; Macquarie University, Australia

## Abstract

Orthodox seeds are living organisms that survive anhydrobiosis and may display dormancy, an inability to germinate at harvest. Seed germination potential can be acquired during a prolonged period of dry storage called after-ripening. The aim of this work was to determine if gene transcription is an underlying regulatory mechanism for dormancy alleviation during after-ripening. To identify changes in gene transcription strictly associated with the acquisition of germination potential but not with storage, we used seed storage at low relative humidity that maintains dormancy as control. Transcriptome profiling was performed using DNA microarray to compare change in gene transcript abundance between dormant (D), after-ripened non-dormant (ND) and after-ripened dormant seeds (control, C). Quantitative real-time polymerase chain reaction (qPCR) was used to confirm gene expression. Comparison between D and ND showed the differential expression of 115 probesets at cut-off values of two-fold change (p<0.05). Comparisons between both D and C with ND in transcript abundance showed that only 13 transcripts, among 115, could be specific to dormancy alleviation. qPCR confirms the expression pattern of these transcripts but without significant variation between conditions. Here we show that sunflower seed dormancy alleviation in the dry state is not related to regulated changes in gene expression.

## Introduction

Seeds contribute to the survival and persistence of domesticated and non-domesticated plants in managed and natural ecosystems. Therefore, the completion of germination represents a key process that permits the maintenance of plant species. Seed germination can be modulated by dormancy, which provides a mechanism for delaying this process to survive unfavorable conditions for seedling establishment. Seed dormancy is defined as the failure of viable mature seeds to germinate under apparently favorable conditions [1]. Dormancy can be caused by the embryo itself and/or tissues surrounding the embryo, specified as embryo dormancy and seed coat imposed dormancy, respectively. Dormancy is released during after-ripening (dry storage), which results in changes in physiological status that induce seed germination [1], [2]. After-ripening is particularly an intriguing phenomenon since the physiological changes that allow the shift from non-permissive to permissive status for germination occur at low moisture content (MC), generally <0.10 g H_2_O/g dry weight (DW) [1], [3]. Under these conditions, water is presumed to be not available for biochemical reactions especially transcription and translation [4]. However, it has been shown that after-ripening trigger gene abundance changes in seeds of *Nicotiana tabacum* [5], *Nicotiana plumbaginifolia* [6], barley [7] and wheat [8]. Leubner-Metzger [5] hypothesized that the presence of hydrated pockets within cells of mature dry seeds would allow an active transcription. However, the possibility of hydrated areas within desiccated tissues is subjected to caution and is not supported by major thermodynamic laws of water equilibrium and binding properties in living tissues [9]. Moreover, the molecular mobility that is required for biochemical reactions is dramatically restricted in dry seeds whose cytoplasm displays a visco-elastic solid state, i.e. a glass [10]. Until now, there has been no experimental evidence supporting or rejecting the occurrence of regulated gene expression during alleviation of seed dormancy in anhydrobiosis, mainly because chemical biology of dry living organs is hardly investigable. In order to bring the first lines of evidence to this unsolved biological question, we have investigated the transcriptomic changes that are likely to occur in dormant dry sunflower seeds (MC close to 0.040 g H_2_O g DW^−1^) and after their storage under 2 relative humidity (RH) regimes, one allowing dormancy alleviation and the other not (60 and 5 % RH, respectively). Such experimental design allows differentiating between change in gene expression associated with the achievement of germination capacity and those associated with storage only.

## Materials and Methods

### Plant Material and After-ripening Treatments

Sunflower (*Helianthus annuus* cv LG5665) seeds produced in the year 2010 in “Drôme” (region of France) were purchased from Limagrain. After harvest, seeds are dormant (D), and they were stored immediately at −23°C until further use in order to maintain their dormancy. Dormant seeds were also stored at 20°C and 60 % RH for two months for after-ripening treatment (ND). Seeds used for storage control (C) were stored for the same duration at 20°C in tightly closed jars over saturated solution of ZnCl_2_ that gave RH of 5 % [3], [11].

### Germination Tests

Germination assays were performed with embryos (i.e. seeds without pericarp) at 10°C in darkness on a layer of cotton wool moistened with deionized water in 9 cm Petri dishes (25 seeds per dish, four replicates). An embryo was considered as germinated when the radicle was elongated by 1 mm. Germination counts were done over a period of 10 days after imbibition.

### RNA Extraction

Embryonic axes were isolated from dry seeds (D, ND and C) and were frozen in liquid nitrogen and stored at −80°C until subsequent use. For each sample, 25 axes were ground to a fine powder in liquid nitrogen, and total RNA was extracted by a hot phenol procedure as previously described by Oracz et al. [12], according to Verwoerd et al. [13]. RNA concentration was determined at 260 nm using Nanovue spectrophotometer (GE healthcare).

### Affymetrix Array Hybridization

RNA hybridization (two biological and two technical repetitions) was performed using Affymetrix Sunflower Gene WT Chip [14], [15] at the affymetrix platform at INRA-URGV, Evry, France. RNA samples were checked for their integrity on a bioanalyzer (Agilent Technologies, Waldbroon, Germany). One hundred ng of total RNA were used for biotin-labelled cRNA synthesis using Genechip® WT cDNA synthesis and amplification kit (Affymetrix, Santa Clara, CA) following the manufacturer’s instructions. The raw and normalized microarray data are available in the database (AFFY_Dormance_totalRNA_Sunflower, Gagnot et al. [16]) and the Gene Expression Omnibus (GEO) repository of the National Center for Biotechnology Information (NCBI), accession number GSE23046 [17].

### Data Analysis

The data were normalized with the Robust Multi-array Average algorithm [18]. Two group t-test was performed to determine differential expression between genes. The raw P values were adjusted by the Bonferroni method, which controls the Family Wise Error Rate [19]. A Bonferroni P-Value less than 0.05 was required for significant variation in gene expression.

### Real-time Quantitative RT–PCR

Total RNA (4 µg) was treated with DNase I (Sigma), reverse transcribed with Revertaid Reverse Transcriptase (Fermentas) in a 25 µl reaction volume and amplified with Mastercycler ep Realplex (Eppendorf) using 5 µl of 30-fold diluted cDNA solution. Primers were designed with primer3 software and their respective sequences are shown in Table 1. Real-time PCRs were performed with the MaximaTM SYBR Green qPCR Master Mix (Fermentas) and 0.23 mM of each primer (Fermentas) in a 15 µl reaction. Cycle thresholds (Cts) were calculated using the Realplex 2.0 software (Eppendorf). For each plate and each gene, a standard curve made with dilutions of cDNA pools was used to calculate the reaction efficiencies, and the relative expression was calculated according to Hellemans et al. [20] with HaEF1, Haβ-tubulin [12] and HaS19 as reference genes (Table 1). An arbitrary value of 1 was assigned to dormant seed samples, which were used as control samples for normalization [21]. Results presented are the means ± SD of three biological replicates.

**Table 1:** Primer sequences of genes used in the present work.

sunflower chips’s genereference	Heliagene accession number(www.heliagene.org)	Primer sequences
Heli060118_st	HuCL00003C004	Left:AAAAGCCAATGCTTACATAACAA
		Right: GAACTCGTGATTCAGGGCTAG
Heli052463_st	HuCL00117C002	Left:TATCGGGAAAATCGGTGA
		Right: GAGGGGAAGTGGTGTGTGTT
Heli058062_st	HuCL21481C001	Left: CCATAGGAGCCAAAAAGCAC
		Right: GCATAGCAGTCGGGAAGAAA
Heli093833_st	HuCX944051	Left: GTCCCCTTGGGCTATCAACT
		Right: TAGAGGCGGGAAAAGTAGCA
Heli022775_st	HuCL12355C001	Left:TCTCTCGTAAGCAGTCATTTTG
		Right: CAGGGGAAGTCATAGCCAAT
Heli059723_st	HuCL11935C002	Left:TGATGAGCAAATACAGCAATCT
		Right: GGGTGCGAAGAAGAAACCAT
Name of H. annnusL. constitutive gene	CGPDB EST accession(http://cgpdb.ucdavis.edu/)	Primer sequences
HaEF1	QH_CA_Contig2764	Left: TCTCCACTCCTCCAACAC
		Right: CTCAATCACTCGCTACACC
Haβ-tubulin	QH_CA_Contig4019	Left: GGCGTCTACCTTCATTGGT
		Right: TCCATCTCATCCATTCCTTC
HaS19	QHG12B09.yg.ab1	Left:ACACACTCACCCCCACCAC
		Right:GGAAAGCACCAACACCAAG

## Results

### After-ripening Induced Dormancy Breaking is Dependent on Relative Humidity

**Figure 1:**
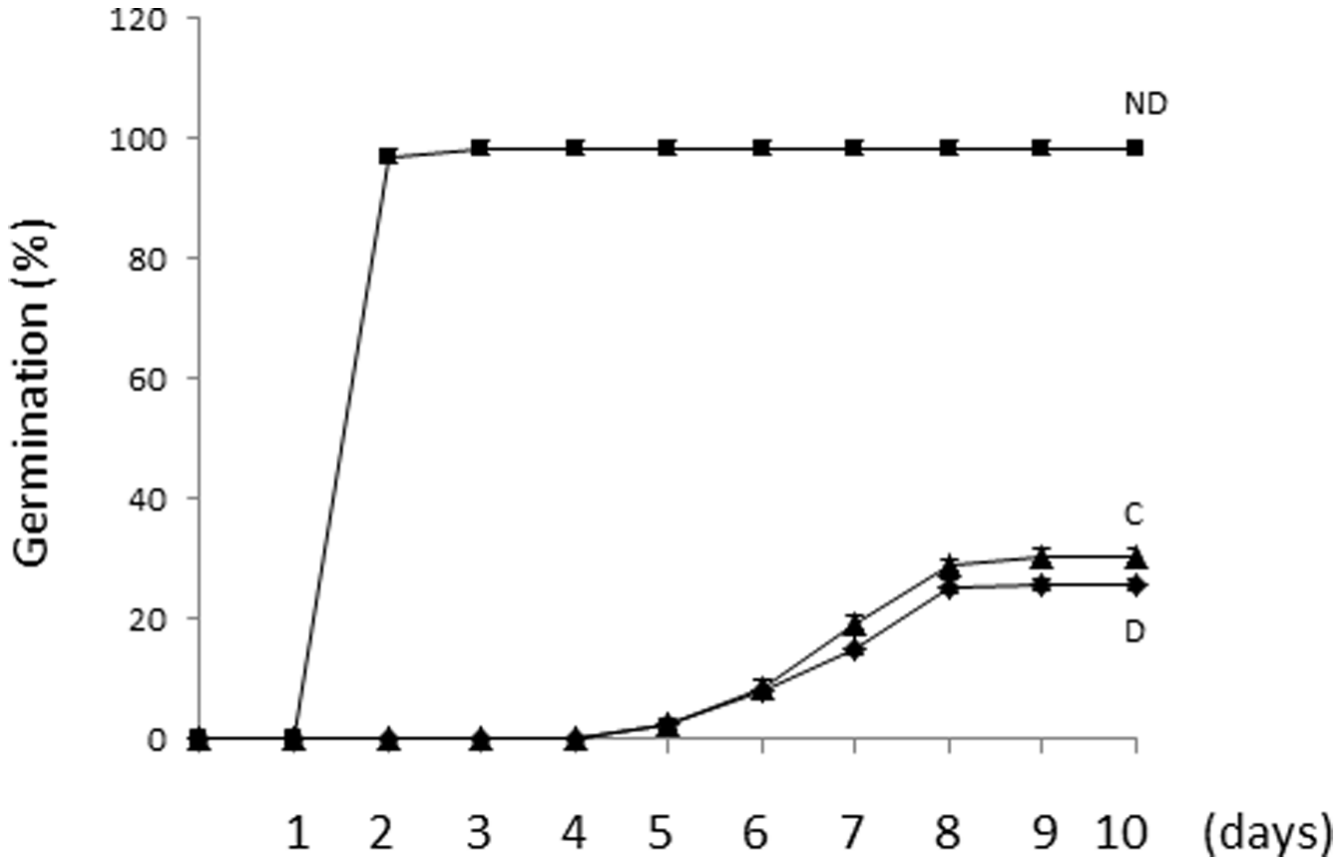
Germination percentage of dormant (D), after-ripened control (C) and non-dormant (ND) seeds.

Figure 1 shows that only 25 % of dormant (D) embryos (after harvest) germinated after 10 days (d) of imbibition at 10°C while 96.6 % of embryos after-ripened at 20°C and 60% RH for 2 months germinated within 2 d at the same temperature (non-dormant, ND, Fig. 1). After 2 months of storage at 5 % RH, only 30.3 % of embryos germinated, demonstrating that low MC prevented dormancy release (storage control, C, Fig. 1).

### Small Change in Expression Profile at Dry State

**Figure 2:**
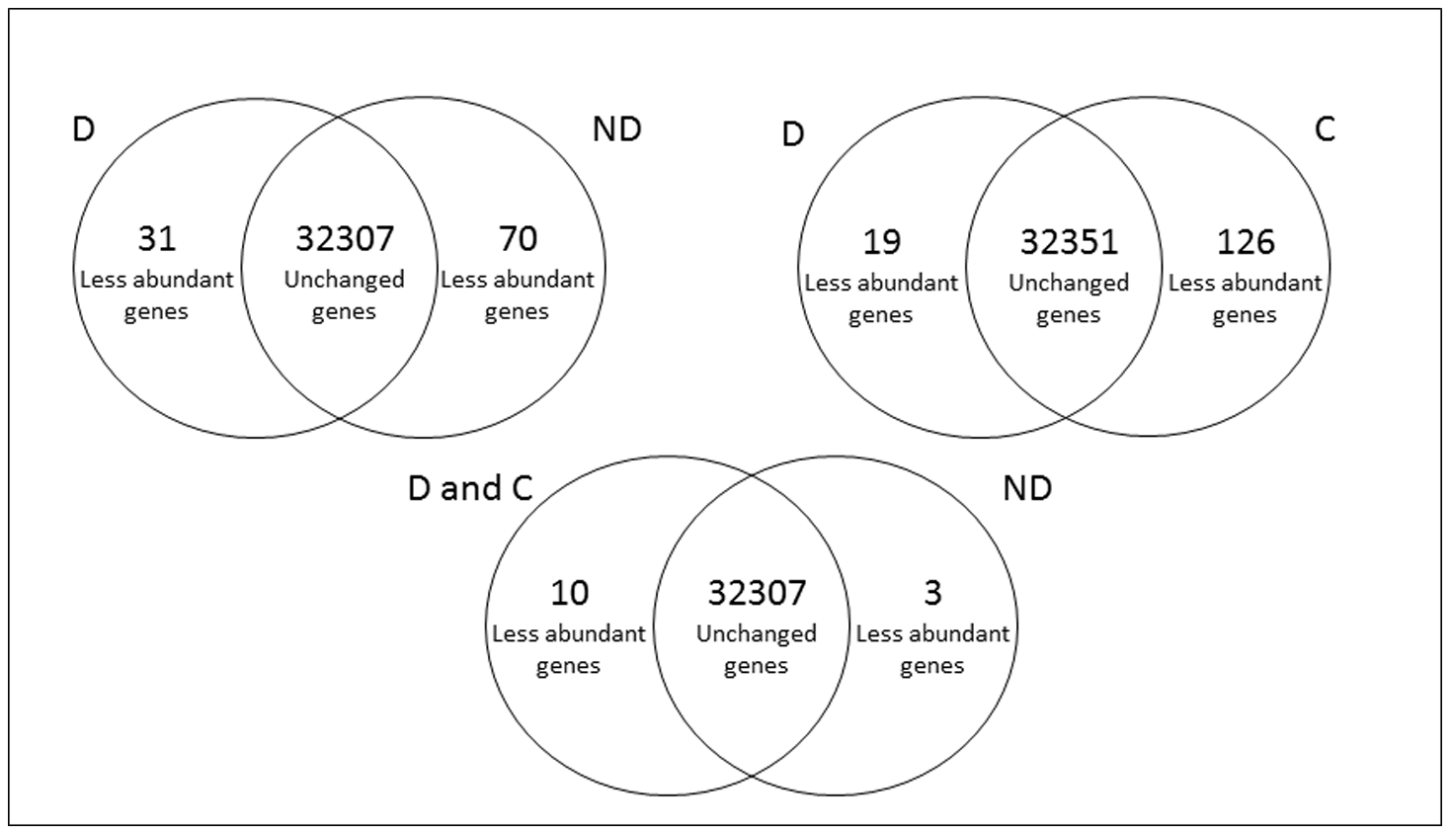
Number of genes with altered expression during after-ripening (≥ 2-fold; p<0.05). Change in gene transcript abundance between dormant (D), after-ripened control (C) and non-dormant (ND) seeds.

We compared mRNA species in dry dormant with after-ripened non-dormant and control embryos using microarray analysis in order to identify dry storage induced transcriptomic changes related to dormancy alleviation. Almost all transcripts represented on the sunflower Affymetrix Genechip^R^ arrays were detected in the embryos from the different conditions. Comparison between D and ND embryos (see Fig. 2) showed that after-ripening triggered differential abundance of 101 probesets at cut-off values of two-fold change (70 less abundant in ND and 31 less abundant in D, Table S1), which represent a small percentage (0.3 %) of total probeset on the Genechips^R^ array. Comparison between D and C samples showed differential abundance of 145 genes (126 less abundant in C and 19 less abundant in D, Table S1). These results show that seed storage was associated with variation in gene expression corresponding mainly to a decrease in transcript abundance.

### Genes Associated with Dormancy Alleviation

**Figure 3:**
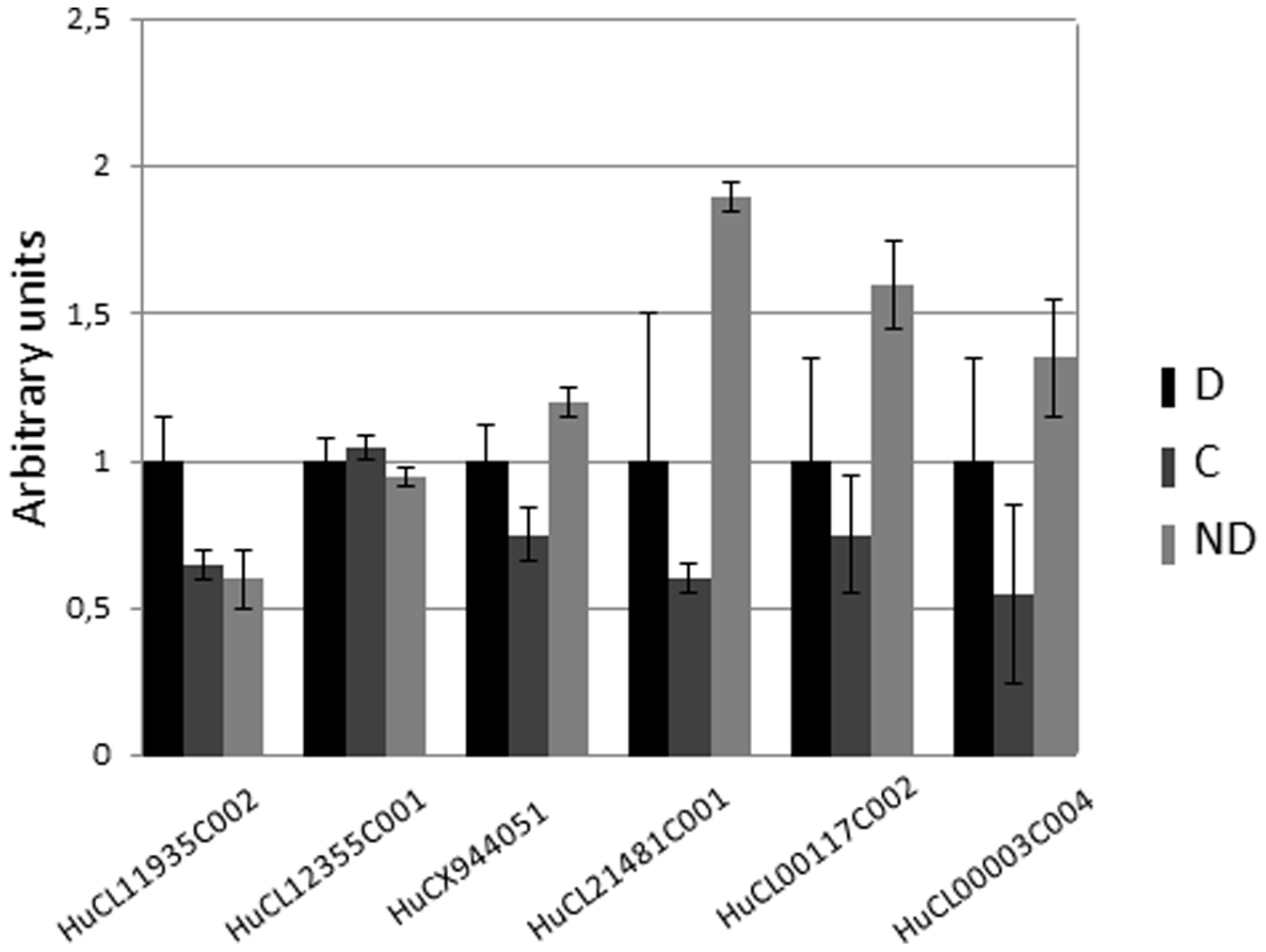
Real-time quantitative PCR analysis of transcripts altered during dry after-ripening. Transcript abundance in after-ripened control (C) and non-dormant (ND) seeds relative to that found in dormant (D) embryos to which the value 1 is assigned. HaEF1, Haβ-tubulin [12] and HaS19 were used as reference genes. Bars represent means ± standard deviation of three biological replicates.

In order to compare changes in gene abundance between dormant (D and C that cannot germinate) and ND (that do germinate), we performed analysis consisting of selected genes that were expressed 2-fold or greater (p<0.05) in both D and C compared with ND. Interestingly, our analysis showed variation of only 13 transcripts, among 115 (Fig. 2). Among the 13 genes with altered transcription, seven show similarity with proteins from other species such as auxin-responsive protein, tify domain containing protein or transport protein particle component (Table 2). Genes whose abundance was altered during after-ripening were studied using real time quantitative PCR. As sequence length of some of the EST did not enable us to design primers for amplification, only 6 of the 13 transcripts were studied. As illustrated in Figure 3, the abundance pattern of these transcripts showed no significant variation between conditions although the pattern was generally confirmed. In fact, HuCL11935C002 was less abundant in ND comparing to D and C but its decrease did not exceed 0.5 fold and the increase in abundance of HuCX944051, HuCL21481C001, HuCL00117C002 and HuCL00003C004 transcripts in ND as compared to D was below 0,5 fold (Fig. 3). For HuCL12355C001, no variation in transcript abundance was observed (Fig. 3). It is important to note that differential abundance of these transcripts was also subtle in the microarray data (Table 2).

**Table 2:** Genes –more or less abundant (≥ 2-fold; p<0.05) in non-dormant (ND) as compared to dormant (D) or after-ripened control (C) sunflower seeds.

Sunflower transcript		Arabidopsishomologue AGI	InterPro domain	Log_2_ (D/ND)	Log_2_ (ND/C)
Affymetrix chip IDwww.heliagene.org	Reference transcriptome IDwww.heliagene.org/HaT13l				
HuCL12355C001	HaT13l000709	ALDH2B4AT3G48000.1	Glutamate-5-semialdehyde dehydrogenase;Aldehyde/histidinoldehydrogenase	−1.74	−1.28
HuCL00003C004	HaT13l002053	ASN1AT3G47340.1	Asparagine synthase, glutamine-hydrolyzingGlutamine amidotransferase,type II	1.7	2.13
HuCD855014	HaT13l003958	AT5G54750.1	Transport protein particle (TRAPP)component; Heme-NO binding	1.11	1.39
HuCX943719	HaT13l008585	alpha/beta-Hydrolasessuperfamily proteinAT4G12230.1	Alpha/beta hydrolase fold-1	1.07	1.06
HuBU029639	HaT13l010420	nd	nd	−1.27	−1.22
HuCL06132C001	HaT13l012873	JAZ6AT1G72450.1	Tify; CO/COL/TOC1, conserved site	1.07	1.12
HuCL00117C002	HaT13l014495	IAA14 (SOLITARY ROOT)AT4G14550.1	AUX/IAA protein	1.23	1.95
HuCL11935C002	HaT13l021935	nd	nd	−1.23	−1.07
HuBU021987	HaT13l031074	TXP2AT5G37478.1	Xklp2 targeting protein	1.11	1.6
HuCL21481C001	HaT13l152236	nd	nd	1.21	1.44
HuCX944051	HaT13l152236	nd	nd	1.14	1.69
HuDY906631	not found	nd	nd	1.12	0.96
HuCX946398	not found	nd	nd	1.11	1.24

nd : not determined.

## Discussion

Sunflower (*Helianthus annuuus* L.) seed displays both seed coat and embryo dormancy [22]. This study concerns embryo dormancy, and thus all experiments were performed with de-coated (naked) sunflower seeds. During after-ripening, sunflower embryo dormancy alleviates but can be modulated by the RH applied (Fig. 1). It is well established that both temperature and seed MC can alter the rate of dormancy release during after-ripening. Here we demonstrated that at a given temperature, MC determines dormancy release. Embryos stored at 5 or 60 % RH at 20 °C displayed a MC at equilibrium at 0.020 and 0.040 g H_2_O g DW^−1^, respectively. It is worth to underline that at such MC, water is considered as being structural and tightly bound on macromolecules [11]. It has been shown that the shift from dormant to non-dormant state occurs within the range of 0.04–0.2 g H_2_O g DW^−1^ [23], [24]. Based on sorption isotherm curve, such range of MC corresponds to weakly bound water [24]. These conditions do not allow biochemical reactions such as gene transcription and translation [4]. However, transcript variation has been reported during dry after-ripening [5], [6], [7], [8]. In this paper, we have used an after-ripening control to distinguish between storage associated- and dormancy alleviation specific- in gene transcription variation.

Transcriptomic analysis showed that almost all transcripts represented on the sunflower Affymetrix Genechip^R^ arrays were detected in the embryos from the different conditions. It has been shown that mature seeds contain transcripts representing over 60 % of the genome of other species as Arabidopsis or rice [25], [26]. It is assumed that non dormant seeds contain stored transcripts ready for use upon imbibition to trigger germination [26].

Only very small percentage (0.3 %) of total probesets represented at a detectable level on the genechip array showed change in expression between dry dormant and after-ripened sunflower seeds. This result is in accordance with that of wheat seeds, as only 58 probesets representing 0.1 % of total probesets detected in wheat affymetrix array have been shown to be differentially present in dry D and after-ripened seeds [8]. These results show that dry after-ripening was associated with very small transcript variation, which rises the question of the occurrence of gene regulation. Finch-savage et al. [27] showed that the expression of all genes represented in Arabidopsis Affymetrix microarray of dry dormant and after-ripened non dormant seeds was similar. On the other hand, for different seed species, variation in gene expression between dormant and after-ripened seeds during imbibition represents a higher number, about 10 fold in wheat for example [8]. The latter proportion is in accordance with gene variation in other physiological processes such as during fruit development [28], response to ionizing radiation [29] or bud dormancy transitioning [30].

Interestingly, variation between D, ND and C transcriptome corresponds mainly to a decrease in transcript abundance (Fig. 2). This trend has been shown in all previous studies that reported changes in transcript abundance during dry after-ripening, using microarrays gene chips or cDNA AFLP [6], [7], [8], [27]. However, an after-ripening storage control has never been used to target genes strictly associated with the acquisition of germination potential. The use of a storage control in the array analysis is important in the field of seed dormancy since it allows ruling out the involvement of changes in gene expression during dry after-ripening. In particular, as it was shown here, seed storage *per se* induces change in transcripts abundance and this change is not related to dormancy alleviation. Consequently, our data suggest that the changes in transcript abundance cannot be related to a putative dormancy alleviation induced gene expression in the dry state. This is in accordance with an early study which reported that genes are not transcribed *in vivo* in dry seeds [31]. Indeed, when dormant seeds were stored at low RH (C), water is supposed to be tightly associated with macromolecules and forming a monolayer [32]. Such structural water is involved in the maintenance of structural integrity of macromolecules and absolutely not available for biochemical reactions, that excludes any transcriptional activity explaining the apparent change in gene expression. This is in accordance with previous indirect evidence, which showed that below an embryo MC of 0.1 g H_2_O g^−1^ DW, sunflower seed dormancy release was associated with negative activation energy corresponding to the occurrence of non-enzymatic reactions rather than metabolic ones [3]. Non-enzymatic reactions, such as lipid peroxidation or the Amadori and Maillard reactions associated with free radical production and oxidation processes are indeed likely to occur during dry storage of seeds. Interestingly, it has been demonstrated that sunflower seed dormancy alleviation is associated with ROS and oxidized products accumulation in embryos, suggesting that ROS might play a major role in dormancy alleviation [33], [34], [14]. Seed stored proteins and mRNA are targets of ROS, since a pool of them became specifically oxidized during after-ripening [34], [14]. It has been shown that mRNA oxidation is associated with dormancy release during after-ripening in sunflower and wheat [14], [35] suggesting cross-species evolutionary conservation of mRNA oxidation as a post-transcriptional seed dormancy-regulating mechanism [35]. Interestingly, mRNA oxidation results in artifacts in cDNA-amplified fragment length polymorphim analysis which may explain apparent expression change reported in dry seeds [14].

Targeted protein and mRNA oxidation and degradation could play a role in the early steps of seed imbibition that govern the process toward germination. Breakdown of specific mRNAs has been implicated in seed dormancy alleviation, and might be a prerequisite to germination [26], [36]. It has been shown that *de novo* transcription is not essential for early stages of germination [37]. Consequently, the selective degradation of negative regulators of cell signaling might represent the major process involved in dormancy alleviation.

The present data strongly support the idea that regulated variation in gene expression does not occur during after-ripening, in agreement with the absence of metabolic activity in dry seeds, and that the apparent transcript abundance changes observed in these conditions are not related to dormancy alleviation. These data and the ones obtained previously [34], [3], [14], allow us to propose that non-enzymatic ROS production is the key event in loss of dormancy during after-ripening in sunflower seeds.

## Supporting Information

Table S1 List of probsets differentially abundant in dry seeds (≥ 2-fold; p<0.05) between dormant (D) and non-dormant (ND; D vs. ND), after-ripened control (C) and ND (C vs. ND) and D and C (D vs. C) samples.
(XLSX)
